# Supplementary figures and images for: Kaposi’s sarcoma-associated herpesvirus latency-associated nuclear antigen dysregulates expression of MCL-1 by targeting FBW7
Source: PLoS Pathog. 2021 Jan 20;17(1):e1009179. doi: 10.1371/journal.ppat.1009179 (PMC7816990; doi:10.1371/journal.ppat.1009179)

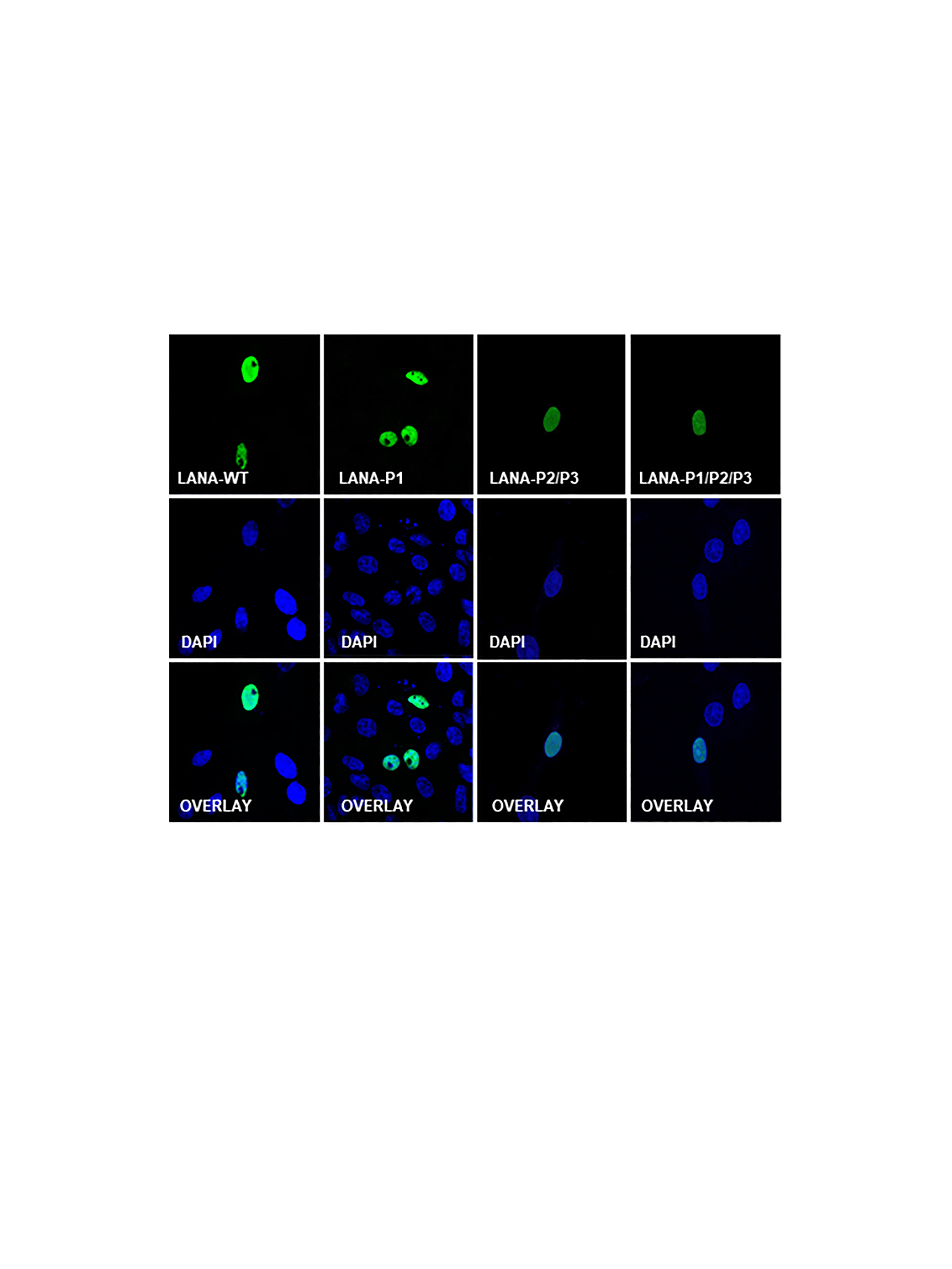

Supplement: S1 Fig — Vero cells transiently expressing LANA wild-type (WT) or three different LANA phospho-dead mutant plasmids were fixed and viewed by confocal microscopy using anti-Au (green) representing LANA. Nuclei (blue) were stained with DAPI. (TIF) [file ppat.1009179.s001.tif]

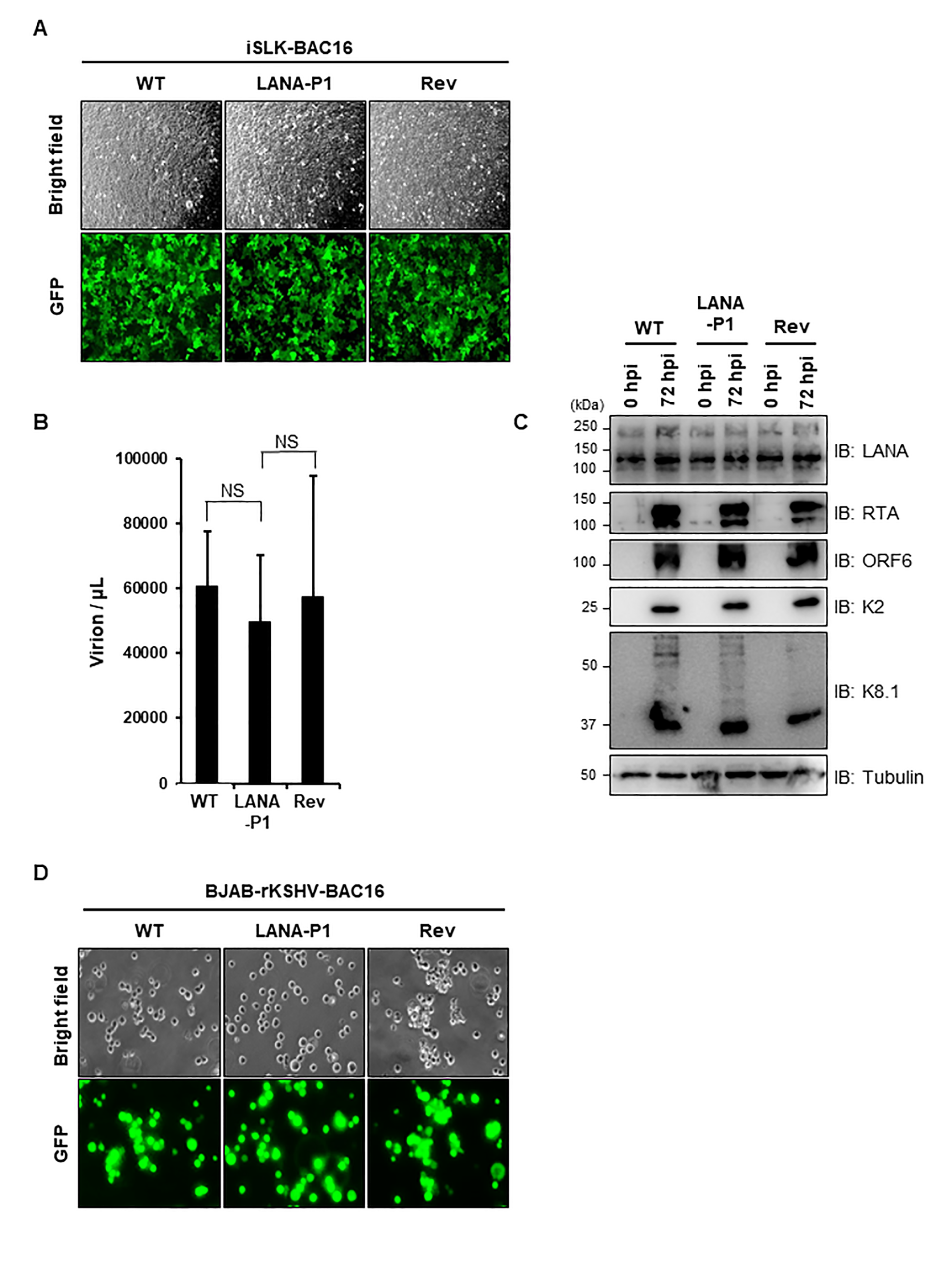

Supplement: S2 Fig — A. Three different iSLK-BAC16 cell lines expressed GFP. B. These iSLK-BAC16 cells were treated with 1 μg/ml of Doxycyline and 1 mM sodium butyrate for 72 hours. After reactivation, virus production was measured at 5 days post-induction based on virion-associated DNA purified from cell culture supernatant. The p- values were calculated applying Student’s two-tailed t-Test. C. The same set of cells were analyzed by immunoblotting using indicated antibodies. Representative IE (RTA), E (ORF6, ORF45, K2), and L (K8.1) viral proteins are shown along with LANA and the loading control tubulin. The average of six biological replicates is shown. D. BJAB cells were co-clutured with reactivated iSLK cells harboring BAC16 KSHV WT, LANA-P1, and Rev. These cells were treated with 200 μg/ml hygromycin B. After establishing BJAB-BAC16 cell lines, cells were monitored for GFP expression in KSHV-BAC16 by fluorescence microscopy. (TIF) [file ppat.1009179.s002.tif]

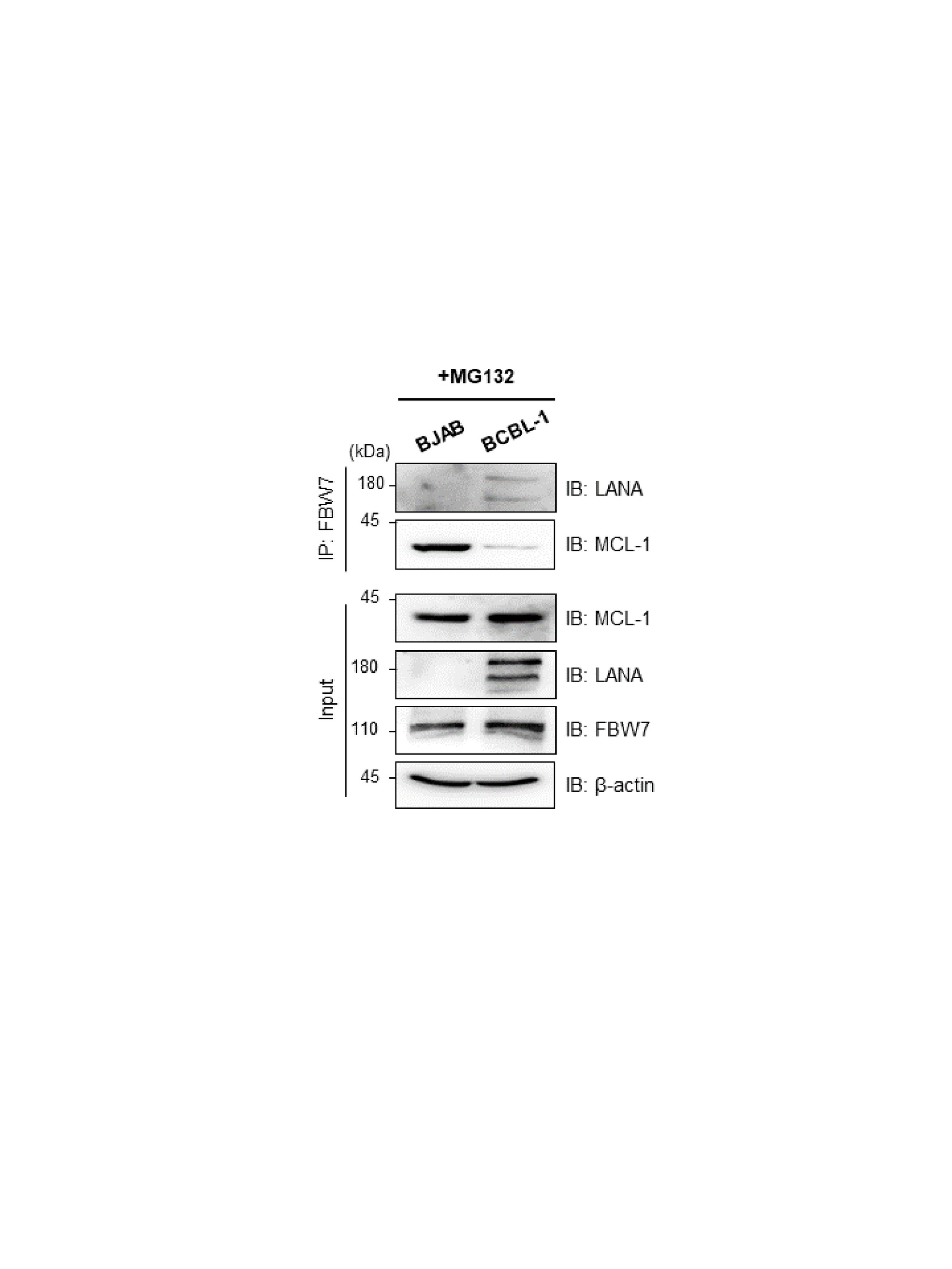

Supplement: S3 Fig — Both BJAB and BCBL-1 cells were treated with MG132 (10 μM) followed by immunoprecipitation with anti-FBW7 antibody and immunoblotting with either an anti-LANA or an anti-MCL-1 antibody. (TIF) [file ppat.1009179.s003.tif]

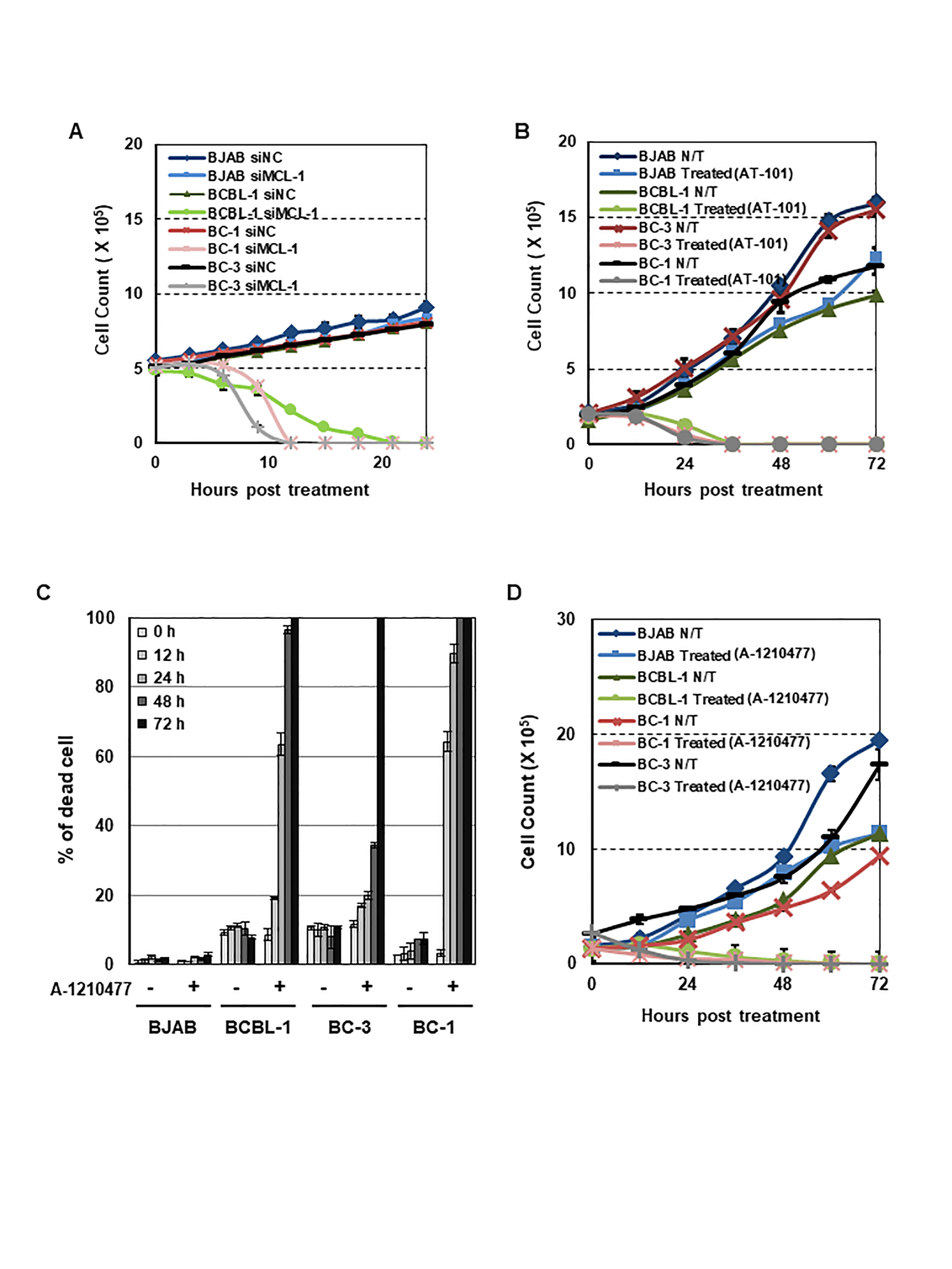

Supplement: S4 Fig — A. BJAB and KSHV-infected PEL cell lines were transfected with negative control siRNA or siRNA that target MCL-1 (100 pmol) for the indicated periods of time, followed by staining with trypan blue solution. Either negative control siRNA- or MCL-1 siRNA-transfected cells were counted for the indicated periods of time. B. AT-101-treated cells were stained with trypan blue solution, followed by counting for the indicated periods of time. Data represent the mean ± SD of the combined results from three independent experiments. C and D. Cells were treated with 10 μM of A-1210477 for the indicated time periods. C, Staining with trypan blue solution; D, Live cells counting. Data represents the means (± SD) of the combined results from three independent experiments. (TIF) [file ppat.1009179.s004.tif]

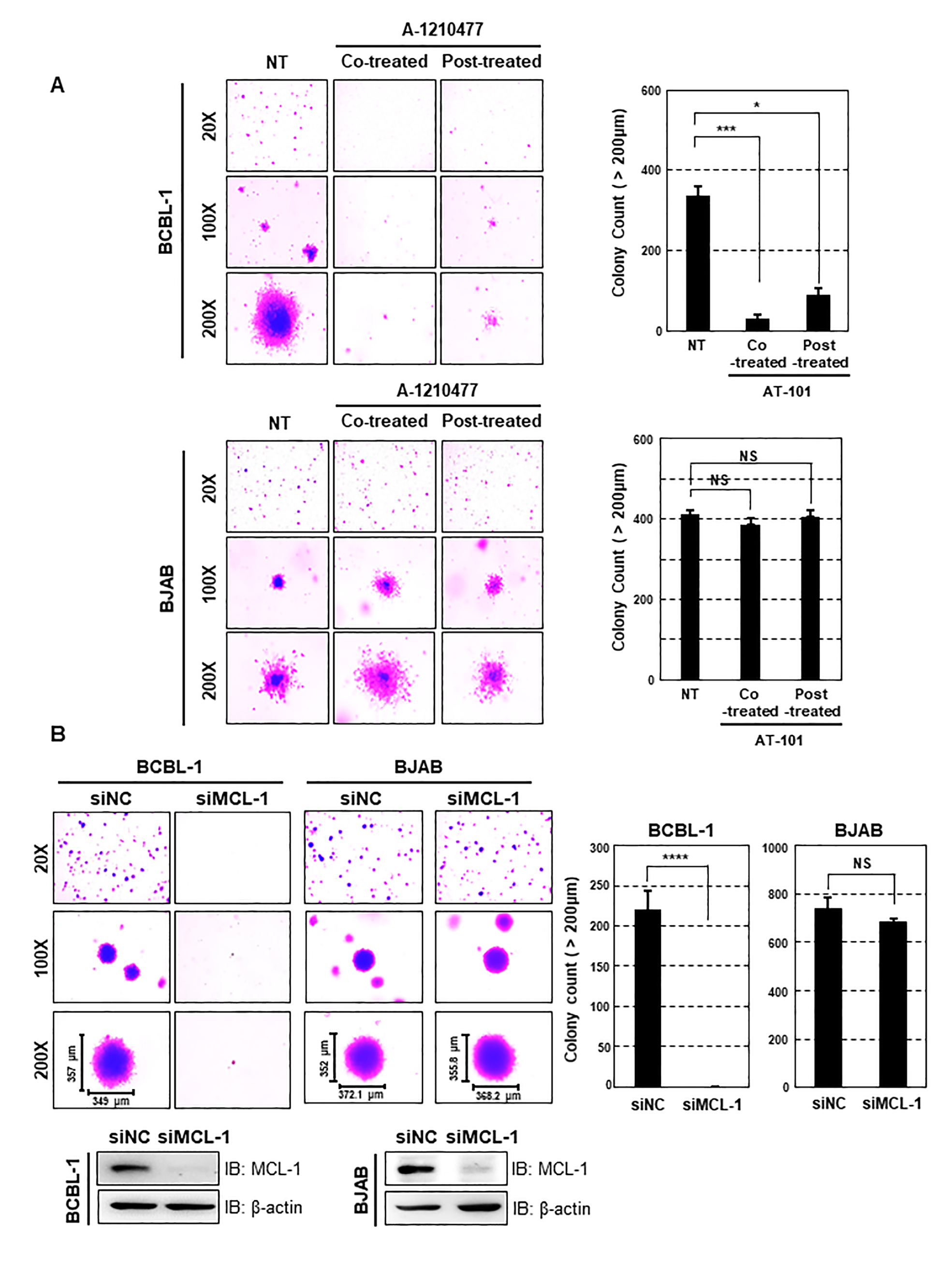

Supplement: S5 Fig — A. BCBL-1 and BJAB cells were plated in soft agar with or without 10 μM of A-1210477 for two weeks. Total colonies larger than 200 μm in representative pictures captured at indicated magnifications were counted. The data represent the means ± SEM. Statistical analysis was performed using two-tailed Student’s t-test. Non significance (NS), p > 0.05; *, p < 0.05; ***, p < 0.0005. B. Negative control siRNA- or siMCL-1-transfected BJAB and BCBL-1 cells were plated in soft agar for 2 weeks (upper). ****, p < 0.00005. Negative control siRNA or siMCL-1-transfected cells were subjected to IB with anti-MCL-1 and anti-β-Actin antibodies (bottom). (TIF) [file ppat.1009179.s005.tif]

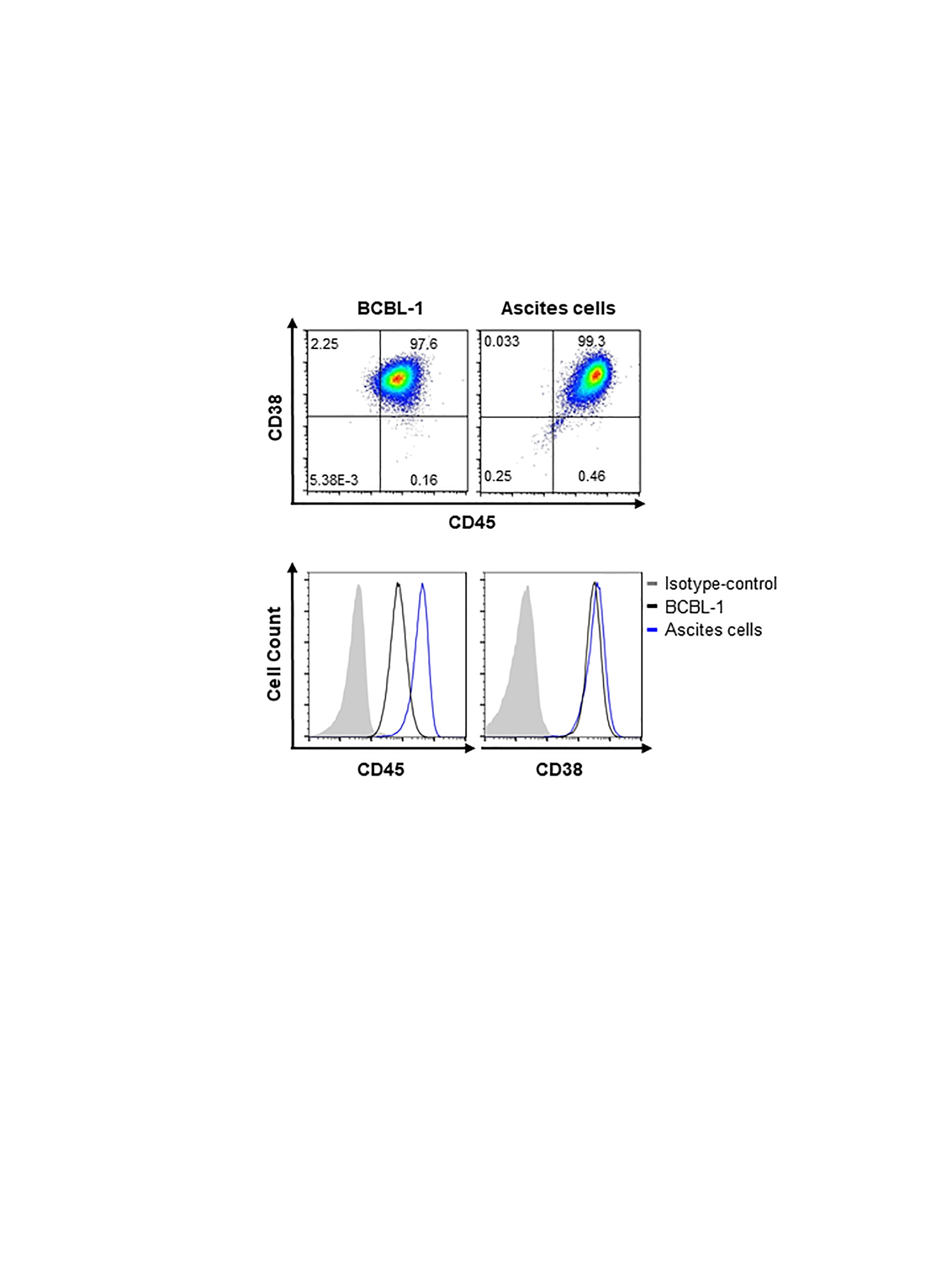

Supplement: S6 Fig — The BCBL-1 cells and cells from ascites fractions were stained for PEL-surface markers CD45 and CD38, and then subjected to FACS analysis. Isotype controls, PE mouse IgG1, κ and mouse IgG1, κ antibodies were used as a negative control for FACS analysis. (TIF) [file ppat.1009179.s006.tif]
