# Supplementary material for: Kaposi’s sarcoma-associated herpesvirus latency-associated nuclear antigen dysregulates expression of MCL-1 by targeting FBW7
Source: PLoS Pathog. 2021 Jan 20;17(1):e1009179. doi: 10.1371/journal.ppat.1009179 (PMC7816990; doi:10.1371/journal.ppat.1009179)
Supplement: Attachment — Submitted filename: Comments for the authors.docx [file ppat.1009179.s007.docx]

Comments for the authors

Myeloid cell leukemia 1 (MCL-1) is an anti-apoptotic protein that is critical for survival of immune cells including T lymphocytes, B lymphocytes, and macrophages. Recently, MCL-1 has emerged also as an essential factor for the survival of KSHV-infected primary effusion lymphoma (PEL) cell lines. Here, the paper re-invited the essential role of MCL-1 in KSHV infection using MCL-1 depletion or inhibitors in cultured PEL cell lines and a xenograft mouse model of PEL. As an original part of the manuscript, the authors surveyed the molecular mechanism by which MCL-1 is stabilized in the PEL cell lines and proposes that LANA competes with MCL-1 for binding to the ubiquitin E3 ligase FBW7, thus reducing ubiquitination and degradation of MCL-1. This proposed model is supported by the use of the LANA mutant P1, identified by the authors, which is incapable of binding to FBW7. While the novelty of the paper is mainly represented by the molecular mechanism of action for LANA-mediated MCL-1 stabilization, key pieces of evidence are still missing, in particular, in the context of virus infection, and the results of polyubiquitination of MCL-1 is not convincing. In addition, the paper lacks necessary discussion on already published LANA interaction with FBW7 and on how the mitochondrial protein MCL-1 is regulated by the nuclear proteins LANA and FBW7.

Specific points that should be addressed:

1. LANA-mediated MCL-1 stabilization and related mechanisms were proposed mainly based on the studies using sole expression of LANA in KSHV-negative BJAB cell line and 293T cells. So, it is not appreciated how important LANA, in particular, via sequestering FBW7, contributes to the elevated expression of MCL-1 in KSHV-infected PEL cell lines as shown in Figure 5A. To address this point, it is desirable to use recombinant KSHV viruses, e.g., BAC16, harboring LANA wild-type (WT), knockout, and revertant with WT or the P1 mutant, for MCL-1 assays in BJAB cells. If applicable, it’d be also nice to identify and use a competitive inhibitory peptide that can block the interaction between LANA and FBW7 in KSHV-infected PEL cells.

2. The paper examined protein interactions using immunoprecipitation of ‘overexpressed’ proteins. Despite the presence of solid evidence, the interactions among LANA, FBW7, and MCL-1 should be further investigated in terms of biological relevance, e.g., i) following etoposide treatment in the BJAB cells expressing LANA WT or P1 and ii) in the PEL cell lines along with control cell BJAB. In either case, MG132 treatment may be needed to preserve MCL-1 protein to make the level of MCL-1 comparable as shown Figure 5B.

3. It is not convincing that the ubiquitinated bands shown in Figures 3B and 5B represent polyubiquitination of ‘MCL-1’. The paper did not describe how MCL-1-associated proteins, probably also ubiquitinated, were removed from the MCL-1-immunoprecipitated complex in the Materials and Methods section. Sonication could not remove MCL-1 interactions with other cellular proteins. In addition, in Figure 3B, controls are missing such as transfection of MCL-1 and HA-Ub or HA-Ub alone.

An increase on polyubiquitination of MCL-1 does not necessarily implicate enhanced proteasomal degradation of MCL-1. Thus, it is desirable to identify what type of polyubiquitination, e.g., K48 and K63, of MCL-1 in the Figures and to examine whether LANA can suppress K48-linked polyubiquitination, which is involved in proteasomal degradation, of MCL-1.

4. In fact, the interaction of LANA and FBW7 (also known as SEL10) was previously reported by Lan K, *et al*. in PNAS (2007), who demonstrated that the oncogenic factor intracellular activated notch (ICN) could be stabilized by LANA competition with FBW7. ICN is a reminiscence of MCL-1. However, both studies show the discrepant results in FBW7-binding regions in LANA. It needs to be addressed. It is curious why ICN was not involved in Figure 2B.

5. MCL-1 is mainly detected and functional in mitochondria. How can the nuclear proteins LANA and FBW7 (Figure 1F) stabilize the mitochondrial protein MCL-1?

6. It is desirable to show the levels of primary (target) proteins, in addition to binding proteins, immunoprecipitated in Figures 1B, 1C, 1D, 1E, 3A, 3B, and 5B.

7. In the top panels of Figures 3B and 5B, how do the authors know that the bottom bands are ‘unmodified MCL-1’? It is unlikely to detect the MCL-1 bands with anti-HA or ubiquitin antibody. Is it possible that the bands are the heavy chain of IgG used in immunoprecipitation?

8. In lines 228-229, the statement, “LANA enhanced endogenous MCL-1 protein levels (Fig. 4B)”, should be rephrased; for example, LANA protected MCL-1 from etoposide-induced degradation. However, ratios of the MCL-1 proteins before and after etoposide treatment look similar among vector, LANA, and LANA-P1 cells. Thus, it may be unlikely that LANA protected MCL-1 from etoposide-induced degradation. It’d be helpful to show the relative band intensities of MCL-1 of each etoposide-treated cell compared to no etoposide treatment.

9. It’d be nice to show the levels of FBW7 in BJAB and the PEL cell lines in Figure 5B.

10. How many mice were used in each group of the xenograft experiment? In Figure 7B, does the number of dots in ascites volume represent the number of sacrificed mice? Detailed description and statistic justification of the animal experiments should be addressed.
