# Supplementary material for: Kaposi’s sarcoma-associated herpesvirus latency-associated nuclear antigen dysregulates expression of MCL-1 by targeting FBW7
Source: PLoS Pathog. 2021 Jan 20;17(1):e1009179. doi: 10.1371/journal.ppat.1009179 (PMC7816990; doi:10.1371/journal.ppat.1009179)
Supplement: Attachment — Submitted filename: Response to Reviewers-final.docx [file ppat.1009179.s008.docx]

**Response to Reviewers**

**Reviewer #1**

**General comment to author:**

Myeloid cell leukemia 1 (MCL-1) is an anti-apoptotic protein that is critical for survival of immune cells including T lymphocytes, B lymphocytes, and macrophages. Recently, MCL-1 has emerged also as an essential factor for the survival of KSHV-infected primary effusion lymphoma (PEL) cell lines. Here, the paper re-invited the essential role of MCL-1 in KSHV infection using MCL-1 depletion or inhibitors in cultured PEL cell lines and a xenograft mouse model of PEL. As an original part of the manuscript, the authors surveyed the molecular mechanism by which MCL-1 is stabilized in the PEL cell lines and proposes that LANA competes with MCL-1 for binding to the ubiquitin E3 ligase FBW7, thus reducing ubiquitination and degradation of MCL-1. This proposed model is supported by the use of the LANA mutant P1, identified by the authors, which is incapable of binding to FBW7. While the novelty of the paper is mainly represented by the molecular mechanism of action for LANA-mediated MCL-1 stabilization, key pieces of evidence are still missing, in particular, in the context of virus infection, and the results of polyubiquitination of MCL-1 is not convincing. In addition, the paper lacks necessary discussion on already published LANA interaction with FBW7 and on how the mitochondrial protein MCL-1 is regulated by the nuclear proteins LANA and FBW7.

**Response to Reviewer #1**

We greatly appreciate reviewer #1 for providing constructive and encouraging comments on our study, enabling us to improve the manuscript by revising it. As suggested by reviewer #1, we have revised the manuscript by including a number of new results. These changes are summarized as follows:

***Major Issues:***

**Q1.** LANA-mediated MCL-1 stabilization and related mechanisms were proposed mainly based on the studies using sole expression of LANA in KSHV-negative BJAB cell line and 293T cells. So, it is not appreciated how important LANA, in particular, via sequestering FBW7, contributes to the elevated expression of MCL-1 in KSHV-infected PEL cell lines as shown in Figure 5A. To address this point, it is desirable to use recombinant KSHV viruses, e.g., BAC16, harboring LANA wild-type (WT), knockout, and revertant with WT or the P1 mutant, for MCL-1 assays in BJAB cells. If applicable, it’d be also nice to identify and use a competitive inhibitory peptide that can block the interaction between LANA and FBW7 in KSHV-infected PEL cells.

**Answer:** We thank reviewer #1 for this insightful comment. As suggested by reviewer, to examine whether LANA has the ability to induce MCL-1 stabilization in KSHV-infected cells, we generated BJAB cell lines with recombinant KSHV (rKSHV)-BAC16 (WT), rKSHV-BAC16-LANA-P1 (LANA-P1), and rKSHV-BAC16-revertant (Rev). Cell lysates were immunoblotted with the indicated antibodies (please refer to Fig. 5B on the right). Our results showed that MCL-1 is highly accumulated in both BJAB-rKSHV-BAC16 and BJAB-rKSHV-BAC16-Rev, but not in BJAB-rKSHV-BAC16-LANA-P1. Furthermore, MCL-1 stabilized by rKSHV infection dramatically suppressed apoptosis by propidium iodide (PI) staining, while increased cell proliferation (see the Fig. 5C and 5D on the above). We have included these results in Fig. 5 of the revised manuscript.

**Q2.** The paper examined protein interactions using immunoprecipitation of ‘overexpressed’ proteins. Despite the presence of solid evidence, the interactions among LANA, FBW7, and MCL-1 should be further investigated in terms of biological relevance, e.g., i) following etoposide treatment in the BJAB cells expressing LANA WT or P1 and ii) in the PEL cell lines along with control cell BJAB. In either case, MG132 treatment may be needed to preserve MCL-1 protein to make the level of MCL-1 comparable as shown Figure 5B.

**Answer:** We sincerely appreciated reviewer’s comment. To validate whether LANA sequesters FBW7 from MCL-1 upon etoposide treatment in terms of biological relevance, TREx/BJAB- vector, -Au-LANA, and -Au-LANA-P1 cells were treated with Doxycycline in the presence or absence of etoposide together with MG132. We performed endogenous FBW7 immunoprecipitation assay and immunoblotted with anti-Au and anti-MCL-1. Our results show that LANA competes with endogenous MCL-1 for binding to endogenous FBW7 (please refer to the Fig. 4C). Moreover, we further confirmed that LANA sequesters FBW7 from MCL-1 in BCBL-1 cells, resulting in reduced FBW7-MCL-1 interaction compared to KSHV^-^ BJAB cells. (See the supplementary Fig. S3). We have included these results in the revised manuscript.

**Q3.** It is not convincing that the ubiquitinated bands shown in Figures 3B and 5B represent polyubiquitination of ‘MCL-1’. The paper did not describe how MCL-1-associated proteins, probably also ubiquitinated, were removed from the MCL-1-immunoprecipitated complex in the Materials and Methods section. Sonication could not remove MCL-1 interactions with other cellular proteins.

In addition, in Figure 3B, controls are missing such as transfection of MCL-1 and HA-Ub or HA-Ub alone. An increase on polyubiquitination of MCL-1 does not necessarily implicate enhanced proteasomal degradation of MCL-1. Thus, it is desirable to identify what type of polyubiquitination, e.g., K48 and K63, of MCL-1 in the Figures and to examine whether LANA can suppress K48-linked polyubiquitination, which is involved in proteasomal degradation, of MCL-1.

**Answer:** We thank for the suggestions.

- As suggested by the reviewer, we carried out the FBW7-mediated ubiquitination of MCL-1 including controls like transfection of MCL-1 alone or MCL-1 and HA-Ub (see the figure on the right). In order to detect ubiquitination specifically on MCL-1 protein, not on non-covalently interacting proteins, we used a stringent condition for cell lysis, immunoprecipitation, and washing that have also been used in shown other studies. Moreover, to prove that the ubiquitin bands shown in Fig. 3B serve as polyubiquitination of MCL-1, we stripped the blot to eliminate the anti-HA antibody (represent overexpressed HA-ubiquitin) and were reprobed with anti-Myc antibody (please see the figure on the above). The band patterns of HA-ubiquitin and 6xMyc (represent MCL-1) immunoblots are correlated with each other, suggesting that the ubiquitin bands shown in Fig. 3B represent polyubiquitination of MCL-1. After rerun the SDS-PAGE gel longer to show better polyubiquitination chain, we performed the immunoblot assay. We replaced Fig. 3B in the revised manuscript.

- In order to verify whether MCL-1 can indeed undergo K48-linked polyubiquitination which LANA can suppress, we carried out an ubiquitination assay in the presence of either wild-type ubiquitin or K48R ubiquitin mutant. Consistent with previous report (*Nature 2011 471, 104-109*), we found that ubiquitination of MCL-1 relies on the presence of FBW7, and LANA dramatically decreased MCL-1 ubiquitination (See Fig. 3B upper panel). However, using the K48R ubiquitin mutant, MCL-1 polyubiquitination was marginally increased upon FBW7 expression, which was not affected by either LANA or LANA-P1 expression (See Fig. 3B lower panel). This result indicates that MCL-1 is K48-linked ubiquitinated, which LANA can suppress, leading to the stabilization of MCL-1. We have changed the Fig. 3B in the revised manuscript.

**Q4.** In fact, the interaction of LANA and FBW7 (also known as SEL10) was previously reported by Lan K, et al. in PNAS (2007), who demonstrated that the oncogenic factor intracellular activated notch (ICN) could be stabilized by LANA competition with FBW7. ICN is a reminiscence of MCL-1. However, both studies show the discrepant results in FBW7-binding regions in LANA. It needs to be addressed. It is curious why ICN was not involved in Figure 2B.

**Answer:** We sincerely appreciate reviewer’s comment. TREx/BJAB vector, LANA, LANA-P1 cells were treated with Doxycycline together with cycloheximide for various time periods and then harvested for immunoblot analysis. In consistent with previous study, our results showed that both LANA and LANA-P1 accumulated ICN, while LANA, but not LANA-P1, stabilized MCL-1 (see Fig. 2B on the right). We also showed that LANA-FBW7 interaction occurs in a phosphorylation dependent manner in Fig. 1. Taken together, our results indicated that MCL-1 is primarily stabilized through the binding of phosphorylated LANA to FBW7. However, expression level of ICN is mainly controlled by interaction between FBW7 and C-terminal region of LANA, but not the phosphorylation dependent manner. We also added immunoblot result of ICN in Fig. 2B of the revised manuscript.

***Minor Issues:***

**Q1.** MCL-1 is mainly detected and functional in mitochondria. How can the nuclear proteins LANA and FBW7 (Figure 1F) stabilize the mitochondrial protein MCL-1?

**Answer:** We thank reviewer #1 for this thoughtful comment. To elucidate how the nuclear proteins LANA and FBW7 stabilize the mitochondrial protein MCL-1, we performed a subcellular fractionation assay using tetracycline-inducible TREx/BJAB cell lines that ectopically expressed Au-tagged LANA or LANA-P1. Interestingly, we found that expression of LANA significantly elevated the nuclear localization of FBW7 compared to vector or LANA-P1 expression, while highly increased MCL-1 level in the mitochondria (see the figure on the above). Taken together, these data indicate that LANA sequesters FBW7 into the nucleus, which can impair the FBW7-mediated ubiquitination of MCL-1 in mitochondria, thereby resulting in accumulation of MCL-1. We have included this result in Fig. 3C.

**Q2.** It is desirable to show the levels of primary (target) proteins, in addition to binding proteins, immunoprecipitated in Figures 1B, 1C, 1D, 1E, 3A, 3B, and 5B

**Answer:** We have added inputs in the revised manuscript. Please see the revised manuscript.

**Q3.** In the top panels of Figures 3B and 5B, how do the authors know that the bottom bands are ‘unmodified MCL-1’? It is unlikely to detect the MCL-1 bands with anti-HA or ubiquitin antibody. Is it possible that the bands are the heavy chain of IgG used in immunoprecipitation?

**Answer:** We sincerely appreciate reviewer’s comment. In Fig. 3B, cells were co-transfected with LANA WT or P1 mutant with FBW7, MCL-1, and HA-ubiquitin. The cells were then treated with MG132, followed by immunoprecipitation with an anti-Myc antibody and immunoblot with an anti-HA (see the figure on the right). The anti-HA antibody was conjugated with HRP, which eliminates the recognition of anti-Myc antibody heavy chain. We then stripped the blot to eliminate the anti-HA antibody and reprobed it with anti-Myc antibody (please see the figure on the right). IgG heavy chain is approximately 45 kDa, 6xMyc-MCL-1 is 60 kDa, and while bands above 60 kDa correspond to ubiquitinated MCL-1. However, we run the gel longer to better show ubiquitinated MCL-1 in Fig. 3B, which was included in the revised Fig. 3B. We note that 6xMyc is approximately 20 kDa (Nat Commun. 2016;7:10574, Nat Commun. 2012;3:978).

In the revised Fig. 6B, in order to avoid the detection the heavy chain of IgG, we utilized rabbit polyclonal MCL-1 antibody for immunoprecipitation and mouse monoclonal ubiquitin antibody for immunoblot. We can detect the endogenous MCL-1 at around 40 kDa as shown in input blot.

**Q4.** In lines 228-229, the statement, “LANA enhanced endogenous MCL-1 protein levels (Fig. 4B)”, should be rephrased; for example, LANA protected MCL-1 from etoposide-induced degradation. However, ratios of the MCL-1 proteins before and after etoposide treatment look similar among vector, LANA, and LANA-P1 cells. Thus, it may be unlikely that LANA protected MCL-1 from etoposide-induced degradation. It’d be helpful to show the relative band intensities of MCL-1 of each etoposide-treated cell compared to no etoposide treatment.

**Answer:** As suggested by reviewer, we have modified the statement in lines 261 of the revised manuscripts. Additionally, we have included a graph, which shows the relative band intensities of MCL-1 in the presence and absence of etoposide. Please see the figure above and Fig. 4B in the revised manuscript.

**Q5.** It’d be nice to show the levels of FBW7 in BJAB and the PEL cell lines in Figure 5B.

**Answer:** We already showed the levels of FBW7 in BJAB and BCBL-1 cells in Fig. 5B. Nevertheless, we added two more PEL cell lines to show FBW7 expression in multiple KSHV^+^PELs compared to KSHV^-^ B cell lymphoma BJAB cells. Please see the figure on the right and see Fig. 6A in the revised manuscript.

**Q6.** How many mice were used in each group of the xenograft experiment? In Figure 7B, does the number of dots in ascites volume represent the number of sacrificed mice? Detailed description and statistic justification of the animal experiments should be addressed.

**Answer:** We utilized 15 mice per each group. Also, the number of dots in ascites volume represents the number of sacrificed mice. We added detailed description of the animal experiments and statistic justification in the revised manuscript.

**Reviewer #2**

 In this interesting manuscript, Kim et al. describe a novel mechanism of how KSHV LANA stabilizes MCL-1, an anti-apoptotic cellular protein, which has previously been shown by others to be highly expressed in and to be essential for the survival of primary effusion lymphoma (PEL) cells and to be a 'druggable' target to treat PEL in preclinical models. The manuscript by Kim et al. therefore contributes two important aspects to the KSHV literature: (i) it shows that KSHV LANA stabilizes MCL-1 by binding to and thereby diverting the E3 ligase FBW7, which normally downregulates MCL-1; (ii) it describes two further MCL-1 or FBW-7 inhibitors that potently inhibit PEL growth in an in vivo preclinical model and thereby extends a previous report on another MCL-1 inhibitor, S63845, which had been shown by others to markedly inhibit the growth of PEL cells in tissue culture. The experiments described in this very well written manuscript are of high technical quality.

**Response to Reviewer #2**

We truly appreciate reviewer #2 for providing insightful and encouraging comments on our manuscript. As suggested by reviewer #2, we have modified the manuscript, which has considerably improved the manuscript. These changes are summarized as follows:

***Major Issues:*** no major criticisms. In my opinion, no additional experiments are needed to support the conclusions drawn in this manuscript.

***Minor Issues:***

**Q1.** Fig. 1F, text line 161: co-localization of LANA WT and LANA P1 with FBW7: if LANA is transfected into cells in the absence of the KSHV genome, it shows a diffuse nuclear distribution which does not lend itself to co-localization studies. The experiment shown in fig. 1F therefore only shows that the LANA P1 mutant localizes to the nucleus, not that it 'co-localizes' with FBW7 (line 161 in the text). I assume that the authors may only have wanted to make the point that the LANA P1 mutant has a nuclear localization. They should therefore change the wording in the text on line 161.

**Answer:** We sincerely thank. We changed the wording in the text. Please see the revised manuscript.

**Q2.** Fig. 5C bottom panel: the WB for BJAB cells suggests that MCL-1 expression levels in BJAB are similar to those in the three PEL cell lines, in contrast to Fig. 5A, which indicates that MCL-1 levels are higher in PEL cells than in BJAB (as also shown on several occasions in the remainder of the manuscript). Presumably this is due to a longer exposure of the WB for BJAB in Fig. 5C. Comparable exposure times should be used for the WBs shown in figure 5C.

**Answer:** These western blots represent two separate blot images. We repeated the siRNA-mediated depletion of MCL-1 in BJAB and the PEL cell lines for 12 h, followed by immunoblotting with anti-MCL-1 and anti-β-actin antibodies. We replaced the previous siRNA western blot image with the new image in Fig. 6C.

**Q3.** Fig. 5D, text line 256: the text states that AT101 induced cell death in PEL cells but not in BJAB; according to fig. 5D, there is a small increase in the number of dead cells also in BJAB cells. AT1210477 also reduces the growth rate of BJAB in fig. S2D, albeit not to the same extent as in PEL cells. The corresponding statements in the text should be rephrased.

**Answer:** We truly appreciate reviewer’s dedicated point. We revised the statements in the manuscript.

**Q4.** line 306: "one of attempt" - better: "an attempt"

**Answer:** We corrected in the revised manuscript. Thank you.
