# Supplementary material for: Kaposi’s sarcoma-associated herpesvirus latency-associated nuclear antigen dysregulates expression of MCL-1 by targeting FBW7
Source: PLoS Pathog. 2021 Jan 20;17(1):e1009179. doi: 10.1371/journal.ppat.1009179 (PMC7816990; doi:10.1371/journal.ppat.1009179)
Supplement: Attachment — Submitted filename: Comments for the authors on the revised manuscript.docx [file ppat.1009179.s009.docx]

Kim *et al* present a revised manuscript that is considerably strengthened in its demonstration of LANA regulation of MCL-1 in the context of virus-infected cells. The authors have enthusiastically performed ubiquitination assays in response to my comments.

However, the authors did not quite understand the concern (minor Q2) raised on co-immunoprecipitation (co-IP) assays. The comment was to show the amounts of primary (target) proteins immunoprecipitated in Figures 1B, 1C, 1D, 1E, 3A, 3B, and 5B. The level of “co-IPed protein” relies on the amount of protein targeted primarily for immunoprecipitation. Thus, in addition to the input (lysate), the levels of the immunoprecipitated primary proteins should be shown; for example, in Fig. 1E, immunoprecipitated Flag-FBW7 should be presented by immunoblotting with anti-Flag antibody.
